# Nation related participation and performance trends in ‘Norseman Xtreme Triathlon’ from 2006 to 2014

**DOI:** 10.1186/s40064-015-1255-5

**Published:** 2015-09-02

**Authors:** Christoph A. Rüst, Nicola Luigi Bragazzi, Alessio Signori, Michael Stiefel, Thomas Rosemann, Beat Knechtle

**Affiliations:** Institute of Primary Care, University of Zurich, Zurich, Switzerland; Department of Health Sciences (DISSAL), School of Public Health, University of Genoa, Genoa, Italy; Department of Health Sciences (DISSAL), Section of Biostatistics, University of Genoa, Genoa, Italy; Department of Neuroscience, Rehabilitation, Ophthalmology, Genetics, Maternal and Child Health (DINOGMI), Section of Psychiatry, University of Genoa, Genoa, Italy; Department of Neuroscience, Rehabilitation, Ophthalmology, Genetics, Maternal and Child Health (DINOGMI), Section of Neurosurgery, University of Genoa, Genoa, Italy; Facharzt FMH für Allgemeinmedizin, Gesundheitszentrum St. Gallen, Vadianstrasse 26, 9001 St. Gallen, Switzerland

**Keywords:** Swimming, Cycling, Running, Ultra-endurance, Mixed model

## Abstract

We investigated the nation related participation and performance trends in triathletes competing in ‘Norseman Xtreme Triathlon’ between 2006 and 2014 using mixed models, one-way analysis of variance and multi-variate regression analyses. A total of 1594 athletes (139 women and 1455 men) originating from 34 different countries finished the race. Most of the athletes originated from Norway, Germany, Great Britain, Sweden, USA and France. In the mixed model analysis considering all finishers (*n* = 1594), with calendar year, sex and country as independent and overall race time as dependent variable, calendar year (*p* < 0.0001), sex (*p* < 0.0001), country (*p* < 0.0001) and the interaction sex × calendar year (*p* = 0.012) were significant. In the model where overall race time was separated in the three disciplines, we found interactions such as country × discipline (*p* < 0.0001), year × discipline (*p* < 0.0001), sex × discipline (*p* < 0.0001), calendar year × sex (*p* = 0.044), calendar year × sex × discipline (*p* = 0.031). Overall race time decreased every year, above all in the year 2012. Women were slower than men, but women reduced this gender gap year after year and above all in the year 2007 (*p* = 0.001). Athletes from Norway and Germany were faster than those from Great Britain and other countries. Split times of the discipline decreased throughout the years. In particular, the discipline having more impact on overall race time was cycling. Most of the podiums were achieved by Norwegian women and men. For women, the fastest split and transition times were achieved by Norwegian women with exception of the run where German women were faster. Norwegian men were the fastest in split and transition times although French athletes were the fastest in swimming. Across years, the annual three fastest Norwegian women improved in cycling, running, overall race time and transition times but not Norwegian and German men. British men, however, improved running split times and transition times. To summarize, most of the finishers in ‘Norseman Xtreme Triathlon’ originated from Norway and the fastest race times were achieved by Norwegian women and men. Norwegian women improved race times across years but not Norwegian men.

## Background

Ultra-distance triathlon is of increasing popularity (Knechtle et al. 2011; Lepers 2008; Meili et al. 2013). In 1978, a group of 15 athletes started at the shores of Waikiki, Hawaii, USA, for the first time in history in a multi-sports event covering 3.8 km swimming, 180 km cycling and 42.195 km running (The Ironman Story 2015). Three years later, the race moved from Waikiki Beach to Kona on Big Island of Hawaii to become the ‘Ironman Hawaii’. Since then, the race is held as the World Championship in Ironman triathlon and the most famous Ironman triathlon (Ironman World Championship 2015).

Nowadays, the ‘Ironman Hawaii’ is considered as one of the 12 toughest sports events in the world (The 12 hardest races in the world 2015). However, in 2003, ‘Norseman Xtreme Triathlon’ was held for the first time in Norway (Norseman Xtreme Triathlon 2015). The first race took place in 2003 with 21 individuals at the starting line. Similarly to ‘Ironman Hawaii’, the ‘Norseman Xtreme Triathlon’ is considered as one of the toughest races in the world (Triathlon Informer 2014). In contrast to the heat and the wind in ‘Ironman Hawaii’ athletes suffer in ‘Norseman Xtreme Triathlon’ from the cold (Norseman Xtreme Triathlon 2015). On race day in ‘Ironman Hawaii’, water temperature is expected at ~26–27 °C. Air temperatures range from 28 to 35 °C with a humidity at around 90 %. Crosswinds during the cycling split may sometimes get as high as 100 km/h (Ironman World Championship 2015). In contrast to ‘Ironman Hawaii’, water temperature in ‘Norseman Xtreme Triathlon’ is at ~15.5–17.5 °C, air temperature on the cycling split at ~5–20 °C, and air temperature on the running split at ~12–28 °C. At the finish at Mountain Gaustatoppen, the air temperature is at ~2–12 °C (Norseman Xtreme Triathlon 2015).

Nationality or belonging to a specific ethnicity seems an important predictor for success in endurance sports (Onywera et al. 2006; Scott et al. 2003; Tucker et al. 2015). For example in running, Kenyan and Ethiopian runners have a distinct environmental background in terms of geographical distribution and ethnicity (Scott et al. 2003; Tucker et al. 2015). For multi-sports events such a triathlon or duathlon, the aspect of nationality has been investigated for Ironman triathletes (Dähler et al. 2014; Jürgens et al. 2012; Stiefel et al. 2013a) and Powerman duathletes (Rüst et al. 2013), but not for Norseman triathletes. ‘Ironman Hawaii’ is dominated by US-American athletes regarding participation and performance although athletes from other countries also showed considerable improvements during 1985 and 2012 (Dähler et al. 2014). Also when ‘Ironman Hawaii’ and its qualifiers were considered for the year 2010, US-American triathletes dominated both participation and performance in both ‘Ironman Hawaii’ and its qualifier races (Stiefel et al. 2013a). Most probably, the proximity of a race leads to the fact that athletes mainly from surrounding countries compete and dominate a specific race. For example, in ‘Ironman Switzerland’ as a qualifier for ‘Ironman Hawaii’, athletes from Switzerland and neighbouring Germany dominated the race (Jürgens et al. 2012). Also when a multi-sports event such as a duathlon is held as a world championship, athletes from the host country and neighbouring countries dominate. In the ‘Powerman Duathlon World Championship’ held in Switzerland, athletes from Switzerland and neighbouring Germany dominated both participation and performance (Rüst et al. 2013). A similar finding was reported for the ‘English Channel Swim’ where most of the participants originated from Great Britain (Knechtle et al. 2014a, b).

To date, no study investigated which athletes dominate ‘Norseman Xtreme Triathlon’. The present study investigated participation and performance trends regarding the nationality of athletes competing in ‘Norseman Xtreme Triathlon’. We investigated the fastest finishers ever by country and the changes in performance across calendar years. Based upon findings for Ironman triathlons (Dähler et al. 2014; Jürgens et al. 2012; Stiefel et al. 2013a), it was hypothesized that ‘Norseman Xtreme Triathlon’ would be dominated by local female and male athletes (i.e. Scandinavian athletes) regarding both participation and performance. Following recent findings from ‘Ironman Hawaii’ (Dähler et al. 2014), we also hypothesized that both female and male athletes would improve their performance across years.

## Methods

### Ethics

The present study was approved by the Institutional Review Board of St. Gallen, Switzerland, with a waiver of the requirement for informed consent given that the study involved the analysis of publicly available data.

### The race

The ‘Norseman Xtreme Triathlon’ is a long-distance triathlon covering the classical Ironman distance of 3.8 km swimming, 180 km cycling and 42.195 km running and is held annually in August in Eidfjord, Norway. The number of participants is limited to 310 entrants (Norseman Xtreme Triathlon 2015). About 40 % of the starters are from outside Norway and approximately 15 % of the starters are women. The ‘Norseman Xtreme Triathlon’ is a point-to-point race with its start in Eidfjord and its finish at Mt. Gaustatoppen (Norseman Xtreme Triathlon 2015).

The swim starts at 05:00 a.m. from the loading bay of a car ferry. The athletes have to jump from the ferry into the fjord and swim then through Hardangerfjord to Eidfjord. In August, the water temperature in Hardangerfjord is generally very low at 13–15 °C (Swimming in cold water 2015). In Eidfjord, the athletes have to change on the bike and then complete the 180 km cycling split through the mountains. The first 40 km of the cycling is uphill reaching 1200 m above sea level. The temperatures during the cycling may vary between 5 and 20 °C. After the finish of the cycling split at Austbygda at 190 m above sea level, the competitors have to run the marathon of 42.2 km of which the first 25 km to Rjukan are flat and following this they end up climbing Mt. Gaustatoppen at 1880 m above sea level (Race manual ‘Norseman Xtreme Triathlon’ et al. 2015). The weather in the mountains can be extreme and may change very quickly to snow, cold and rain. The mean temperature during the run split is 12–28 °C in the valley and 2–12 °C at Mt. Gaustatoppen.

The ‘Norseman Xtreme Triathlon’ is an unsupported race. All competitors need a personal support crew. The crew has to follow the athlete with a car to support with food and drink. The support crew needs to follow the athlete in the final mountain climb since athletes might suffer severe fatigue and problems in the final stage of the race. Severe weather conditions, deadlines and health checks decide whether the race can be held until the official finish. Official finishers of the full distance will get a black T-shirt and are official finishers called ‘Norsemen’. Athletes who finish the race out of the cut-off time on a different route will get a white T-shirt. The cut-off time for the black finisher T-shirt is 14:30 h:min at the 32.5 km mark, and 15:30 h:min at the 37.5 km mark. Only 160 athletes will be permitted to continue from 32.5 km on the black track to Mt. Gaustatoppen under ordinary weather and race conditions. All athletes arriving later will finish on the white track. This means that the 161st athlete to reach 32.5 km will be on the white shirt track even he/she reaches the checkpoint before the cut-off time (Norseman Xtreme Triathlon 2015).

### Data sampling and data analysis

The data set for this study was obtained from the race website of ‘Norseman Xtreme Triathlon’ (Norseman Xtreme Triathlon 2015). Data from 2003 to 2005 were not considered to be reliable and we started data analysis in 2006.

### Statistical analysis

Athletes from all countries with at least ten female or male official finishers were analysed. In a first step, we performed a multi-variate analysis with calendar year, athlete, sex and country as the independent and overall race time (min) as the dependent variable. To find differences between the ten fastest women and men ever per country, a one-way analysis of variance (ANOVA) with subsequent Tukey multiple comparison test with a single pooled variance was used. In order to investigate changes in performance, mixed models were performed, since this sophisticated and refined statistical approach is particularly useful to model the athletic performance from a quantitative point of view (Vandenbogaerde and Hopkins 2010). We used the compound symmetry as co-variance structure, since it assumes the same co-variance between any two or more repeated measurements and the same variance for each measurement. Subjects were considered each athlete and repeated variable each performance. To provide results that could be easily interpreted, different models were built, categorizing the countries in the first top country (Norway) and others (first model), and in the three top countries (Norway, German and Great Britain) and others (second model). The same models were run before and after rescaling the year and using percentiles: in particular, in order to obtain a balanced analysis, the years were clustered in three groups, the first including the years from 2006 to 2008, the second group the years from 2009 to 2011 and the third group the year from 2012 to 2014. This model was built, in order to investigate the impact of each single discipline and its interaction with the other studied variables (namely, year, country, and sex). Total time was decomposed into the split time taken for performing the three disciplines. Subject variable included each athlete, whilst repeated variables included each performance and the discipline. The dependent variable was the total time, whilst the independent variables were the calendar year, the country, the sex, and the discipline. Changes in performance (i.e. split and overall race times) were calculated for the annual top three finishers for all countries with at least three annual finishers. Statistical analyses were performed using IBM SPSS Statistics (Version 22, IBM SPSS, Chicago, IL, USA) and GraphPad Prism (Version 6.01, GraphPad Software, La Jolla, CA, USA). Significance was accepted at *p* < 0.05 (two-sided for *t* tests). Data in the text and figures are given as mean ± standard deviation (SD).

## Results

In the mixed model analysis considering all finishers (*n* = 1594), with calendar year, sex and country as the independent variables and overall race time as the dependent variable, calendar year (*p* < 0.0001), sex (*p* < 0.0001), country (*p* < 0.0001) and the interaction sex × calendar year (*p* = 0.012 in the model with top country and *p* = 0.008 after rescaling the year and using the percentiles; *p* = 0.014 in the model with top three countries and *p* = 0.011 after rescaling the year and using the percentiles) were statistically significant (Tables 1, 2, 3, 4). In particular, overall race time decreased every year, above all in the year 2012. Women were slower than men, but women reduced this gender gap year after year and above all in the year 2007 (*p* = 0.001). Athletes from Norway and Germany were faster than those coming from Great Britain and the other countries.

**Table 1:** Estimation of the fixed effects of the mixed model with the three top countries (Norway or NOR, German or GER and Great Britain or GBR) with overall race time as dependent variable

Parameters	*F*	*p*
Intercept	35,386.32	<0.0001
Year	42.62	<0.0001
Sex	21.07	<0.0001
Country	11.73	<0.0001
Year × sex	4.57	0.011

Calendar year is rescaled using percentiles (Year = 1 is the period from 2006 to 2008, Year = 2 the period from 2009 to 2011, Year = 3 the period from 2012 to 2014). Sex Code = 1 is female, Sex Code = 2 is male

**Table 2:** Results from the mixed model with the three top countries (Norway or NOR, German or GER and Great Britain or GBR) with overall race time as dependent variable

Parameter	Estimated coefficient	SD	*T*	*p*	95 % confidence interval	
					Lower bound	Upper bound
Intercept	867.87	5.27	164.41	<0.0001	857.51	878.22
[Year = 1]	49.98	4.78	10.44	<0.0001	40.60	59.37
[Year = 2]	36.47	4.39	8.30	<0.0001	27.85	45.08
[Year = 3]	0	0	–	–	–	–
[Sex = 1]	17.77	10.59	1.67	0.093	−2.99	38.55
[Sex = 2]	0	0	–	–	–	–
[Country = GBR]	22.34	8.51	2.62	0.009	5.63	39.05
[Country = GER]	−26.55	11.02	−2.40	0.016	−48.17	−4.93
[Country = NOR]	−17.48	5.68	−3.07	0.002	−28.63	−6.33
[Country = other]	0	0	–	–	–	–
[Year = 1] × [Sex = 1]	52.42	17.41	3.01	0.003	18.26	86.59
[Year = 1] × [Sex = 2]	0	0	–	–	–	–
[Year = 2] × [Sex = 1]	12.73	13.96	0.91	0.362	−14.66	40.14
[Year = 2] × [Sex = 2]	0	0	–	–	–	–
[Year = 3] × [Sex = 1]	0	0	–	–	–	–
[Year = 3] × [Sex = 2]	0	0	–	–	–	–

Calendar year is rescaled using percentiles (Year = 1 is the period from 2006 to 2008, Year = 2 the period from 2009 to 2011, Year = 3 the period from 2012 to 2014). Sex Code = 1 is female, Sex Code = 2 is male

**Table 3:** Estimation of the fixed effects of the mixed model

Parameters	*F*	*p*
Intercept	35,236.88	<0.0001
Year	33.58	<0.0001
Sex	21.23	<0.0001
Country	11.88	<0.0001
Discipline	12,359.19	<0.0001
Sex × discipline	12.93	<0.0001
Country × discipline	19.48	<0.0001
Year × discipline	15.19	<0.0001
Year × sex	3.63	0.026
Year × sex × discipline	3.23	0.012

Calendar year has been rescaled using percentiles. Total time has been decomposed into the split time taken for each discipline

**Table 4:** Results from the mixed model with the three top countries (Norway or NOR, German or GER and Great Britain or GBR)

					Lower bound	Upper bound
Intercept	69.87	2.47	28.24	<0.0001	65.02	74.72
[Year = 1]	4.19	2.49	1.68	0.092	−0.68	9.08
[Year = 2]	8.74	2.36	3.69	<0.0001	4.10	13.38
[Year = 3]	0	0	–	–	–	–
[Sex Code = 1]	1.89	5.12	0.37	0.712	−8.15	11.94
[Sex Code = 2]	0	0	–	–	–	–
[Country = GBR]	4.39	3.87	1.13	0.257	−3.19	11.97
[Country = GER]	−1.79	4.97	−0.36	0.718	−11.54	7.95
[Country = NOR]	9.04	2.58	3.49	<0.0001	3.96	14.11
[Country = other]	0	0	–	–	–	–
[Discipline = Bike]	365.90	2.97	122.83	<0.0001	360.06	371.75
[Discipline = Run]	282.68	2.97	94.89	<0.0001	276.84	288.52
[Discipline = Swim]	0	0	–	–	–	–
[Discipline = Bike] × [Sex Code = 1]	14.45	6.24	2.31	0.021	2.21	26.69
[Discipline = Run] × [Sex Code = 1]	−2.86	6.24	−0.45	0.646	−15.10	9.37
[Discipline = Swim] × [Sex Code = 1]	0	0	–	–	–	–
[Discipline = Bike] × [Sex Code = 2]	0	0	–	–	–	–
[Discipline = Run] × [Sex Code = 2]	0	0	–	–	–	–
[Discipline = Swim] × [Sex Code = 2]	0	0	–	–	–	–
[Discipline = Bike] × [Country = GBR]	2.10	4.58	0.46	0.646	−6.88	11.09
[Discipline = Run] × [Country = GBR]	5.84	4.58	1.27	0.202	−3.14	14.83
[Discipline = Swim] × [Country = GBR]	0	0	–	–	–	–
[Discipline = Bike] × [Country = GER]	−12.01	5.87	−2.04	0.041	−23.54	−0.48
[Discipline = Run] × [Country = GER]	−8.40	5.87	−1.43	0.153	−19.93	3.11
[Discipline = Swim] × [Country = GER]	0	0	–	–	–	–
[Discipline = Bike] × [Country = NOR]	−24.19	3.06	−7.89	<0.0001	−30.20	−18.18
[Discipline = Run] × [Country = NOR]	−21.26	3.06	−6.94	<0.0001	−27.27	−15.26
[Discipline = Swim] × [Country = NOR]	0	0	–	–	–	–
[Discipline = Bike] × [Country = other]	0	0	–	–	–	–
[Discipline = Run] × [Country = other]	0	0	–	–	–	–
[Discipline = Swim] × [Country = other]	0	0	–	–	–	–
[Discipline = Bike] × [Year = 1]	29.91	3.07	9.71	<0.0001	23.87	35.95
[Discipline = Run] × [Year = 1]	6.95	3.07	2.25	0.024	0.91	12.99
[Discipline = Swim] × [Year = 1]	0	0	–	–	–	–
[Discipline = Bike] × [Year = 2]	6.58	2.97	2.21	0.027	0.75	12.41
[Discipline = Run] × [Year = 2]	−2.80	2.97	−0.94	0.347	−8.63	3.03
[Discipline = Swim] × [Year = 2]	0	0	–	–	–	–
[Discipline = Bike] × [Year = 3]	0	0	–	–	–	–
[Discipline = Run] × [Year = 3]	0	0	–	–	–	–
[Discipline = Swim] × [Year = 3]	0	0	–	–	–	–
[Year = 1] × [Sex Code = 1]	0.29	8.77	0.03	0.973	−16.91	17.51
[Year = 1] × [Sex Code = 2]	0	0	–	–	–	–
[Year = 2] × [Sex Code = 1]	5.08	7.72	0.65	0.511	−10.07	20.23
[Year = 2] × [Sex Code = 2]	0	0	–	–	–	–
[Year = 3] × [Sex Code = 1]	0	0	–	–	–	–
[Year = 3] × [Sex Code = 2]	0	0	–	–	–	–
[Discipline = Bike] × [Year = 1] × [Sex Code = 1]	17.03	10.69	1.59	0.111	−3.93	38.01
[Discipline = Run] × [Year = 1] × [Sex Code = 1]	32.54	10.69	3.04	0.002	11.56	53.52
[Discipline = Swim] × [Year = 1] × [Sex Code = 1]	0	0	–	–	–	–
[Discipline = Bike] × [Year = 1] × [Sex Code = 2]	0	0	–	–	–	–
[Discipline = Run] × [Year = 1] × [Sex Code = 2]	0	0	–	–	–	–
[Discipline = Swim] × [Year = 1] × [Sex Code = 2]	0	0	–	–	–	–
[Discipline = Bike] × [Year = 2] × [Sex Code = 1]	4.45	9.78	0.45	0.649	−14.72	23.63
[Discipline = Run] × [Year = 2] × [Sex Code = 1]	−4.69	9.78	−0.48	0.631	−23.87	14.48
[Discipline = Swim] × [Year = 2] × [Sex Code = 1]	0	0	–	–	–	–
[Discipline = Bike] × [Year = 2] × [Sex Code = 2]	0	0	–	–	–	–
[Discipline = Run] × [Year = 2] × [Sex Code = 2]	0	0	–	–	–	–
[Discipline = Swim] × [Year = 2] × [Sex Code = 2]	0	0	–	–	–	–
[Discipline = Bike] × [Year = 3] × [Sex Code = 1]	0	0	–	–	–	–
[Discipline = Run] × [Year = 3] × [Sex Code = 1]	0	0	–	–	–	–
[Discipline = Swim] × [Year = 3] × [Sex Code = 1]	0	0	–	–	–	–
[Discipline = Bike] × [Year = 3] × [Sex Code = 2]	0	0	–	–	–	–
[Discipline = Run] × [Year = 3] × [Sex Code = 2]	0	0	–	–	–	–
Discipline = Swim] × [Year = 3] × [Sex Code = 2]	0	0	–	–	–	–

Calendar year is rescaled using percentiles (Year = 1 is the period from 2006 to 2008, Year = 2 the period from 2009 to 2011, Year = 3 the period from 2012 to 2014). Sex Code = 1 is female, Sex Code = 2 is male

In the model in which overall race time was decomposed in the split times of the three disciplines, we found that also interactions such as country × discipline (*p* < 0.0001), year × discipline (*p* < 0.0001), sex × discipline (*p* < 0.0001), calendar year × sex (*p* = 0.044 in the model with the top country and *p* = 0.020 after rescaling the calendar year and using the percentiles; *p* = 0.053 in the model with the three top countries and *p* = 0.026 after rescaling the year and using the percentiles), calendar year × sex × discipline (*p* = 0.031 in the model with the top country and *p* = 0.009 after rescaling the year and using the percentiles; *p* = 0.042 in the model with the three top countries and *p* = 0.012 after rescaling the year and using the percentiles), were statistically significant. Split times of the three disciplines decreased throughout the years. In particular, the discipline having more impact on overall race time was cycling. Considering the interaction between the discipline and the country, whilst this was not statistically significant for Great Britain, it was relevant for German for bike only and for Norway both for bike and run.

### Participation trends

**Fig. 1:**
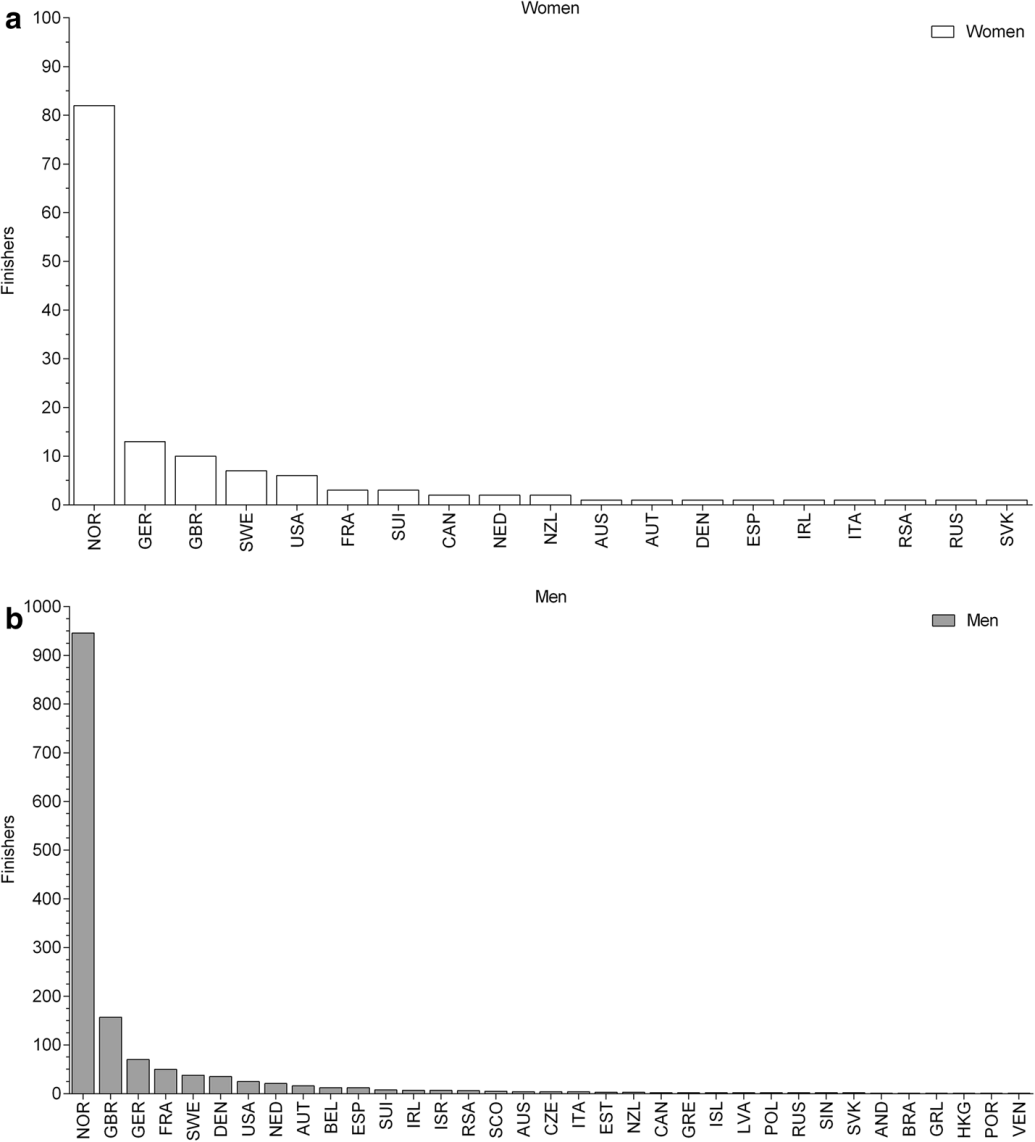
Number of finishers per country for women (**a**) and men (**b**) sorted by frequency. *AND* Andorra, *AUS* Australia, *AUT* Austria, *BEL* Belgium, *BRA* Brazil, *CAN* Canada, *CZE* Czech Republic, *DEN* Denmark, *ESP* Spain, *EST* Estonia, *FRA* France, *GBR* Great Britain, *GER* Germany, *GRE* Greece, *GRL* Greenland, *HKG* Hong Kong, *IRL* Ireland, *ISL* Iceland, *ISR* Israel, *ITA* Italy, *LVA* Latvia, *NED* Netherlands, *NOR* Norway, *NZL* New Zealand, *POL* Poland, *POR* Portugal, *RSA* South Africa, *RUS* Russia, *SIN* Singapore, *SCO* Scotland, *SUI* Switzerland, *SVK* Slovakia, *SWE* Sweden, *USA* United States of America, *VEN* Venezuela

Between 2006 and 2014, a total of 1594 (139 women and 1455 men) athletes finished ‘Norseman Xtreme Triathlon’. The athletes originated from 34 different countries (Fig. 1). Most of the athletes originated from Norway (1028, 82 women and 946 men), Germany (170, 13 women and 157 men), Great Britain (80, 10 women and 70 men), Sweden (57, 7 women and 50 men), USA (44, 6 women and 38 men) and France (38, 3 women and 35 men) (Fig. 1). Considering the number of finishers per country with at least three annual finishers, the number remained unchanged (*p* > 0.05) for women from Norway (9 ± 5, range 5–21) and men from Norway (105 ± 35, range 74–195), Germany (8 ± 3, range 4–14) and Great Britain (17 ± 8, range 7–30).

### Performance trends

Most of the podiums were achieved by Norwegian women and men (Table 5). Considering the ten fastest finishers ever per country, the fastest women originated from Norway, Germany and Great Britain (Table 6). The fastest split and transition times were achieved by Norwegian women with the exception of the run split where German women were the fastest. ANOVA showed no differences between the three countries for swimming. For cycling, Norwegian women were faster than British women (*p* < 0.0001) and German women were faster than British women (*p* < 0.001). Norwegian women were not faster than German women (*p* > 0.05). In running, Norwegian women were faster than British women (*p* < 0.0001) and German women were faster than British women (*p* < 0.05). German women were not faster than Norwegian women (*p* > 0.05). For overall race time, Norwegian women were faster than British women (*p* < 0.0001), but not faster than German women (*p* > 0.05). German women were faster than British women (*p* < 0.001). For transition times, Norwegian women were faster than British women (*p* < 0.01), but not faster than German women (*p* > 0.05). German women w ere faster than British women (*p* < 0.05).

**Table 5:** Top three women and men in ‘Norseman Xtreme Triathlon’ from 2003 to 2014

	1st place	2nd place	3rd place	1st place	2nd place	3rd place
2003	Christian Houge-Thiss (NOR)	Mathias Rasch-Halvorsen (NOR)	Bjørn Wigdel (NOR)	–	–	–
2004	Rune Høydahl (NOR)	Håvard Jensen (NOR)	Christian Houge-Thiss (NOR)	Trude Andersen (NOR)	Solveig Gysland (NOR)	Ingvill Merete Stedøy (NOR)
2005	Björn Andersson (SWE)	Kristofer Larsen (NOR)	Erik Johnsen (NOR)	Trude Andersen (NOR)	Silke Hamacher (NOR)	Sandra Fantini (FRA)
2006	Ole Stougaard (DEN)	Thomas Nesset Sundal (NOR)	Jonas Colting (SWE)	Marie Veslestaul (NOR)	Helene Pallesen (DEN)	Marit Svenning Berg (NOR)
2007	Lars Petter Stormo (NOR)	Romuald Lepers (FRA)	Ole Stougaard (DEN)	Emily Finanger (NOR)	Marit Svenning Berg (NOR)	Lene Hansen (NOR)
2008	Øyvind Johannessen (NOR)	Tom Remman (NOR)	Arild Christophersen (NOR)	Jenny Gowans (GBR)	Heidi Harviken (NOR)	Marie Veslestaul (NOR)
2009	Tom Remman (NOR)	Henrik Oftedal (NOR)	Daniel Blankenfuland (GER)	Susanne Buckenlei (GER)	Vibke Nørstebø (NOR)	Cesilie Skollerud Hegna (NOR)
2010	Henrik Oftedal (NOR)	Tom Remman (NOR)	Even Hersleth (NOR)	Susanne Buckenlei (GER)	Gonny Rosendaal (NED)	Malin Lundvik (SWE)
2011	Tim DeBoom (USA)	Markus Stierli (SUI)	Tom Remman (NOR)	Susanne Buckenlei (GER)	Malin Lundvik (SWE)	Cesilie Skollerud Hegna (NOR)
2012	Henrik Oftedal (NOR)	Lars Petter Stormo (NOR)	Tom Remman (NOR)	Annett Finger (GER)	Charlotte Knudsen (NOR)	Lisbeth Olsen Kenyon (USA)
2013	Markus Stierli (SUI)	Dirk Wijnalda (NED)	Allan Hovda (NOR)	Inger Liv Bjerkreim Nilsen (NOR)	Lydia Waldmüller (AUT)	Line Foss (NOR)
2014	Allan Hovda (NOR)	Lars Christian Vold (NOR)	Per Morten Ellingsen (NOR)	Line Foss (NOR)	Maggie Rusch (USA)	Trude Gran (NOR)

*AUT* Austria, *DEN* Denmark, *FRA* France, *GER* Germany, *GBR* Great Britain, *NOR* Norway, *SUI* Switzerland, *SWE* Sweden, *USA* United States of America

**Table 6:** Split times, overall race times and transition times for the ten fastest women and men ever, sorted by the number of finishers per country

Country	Swimming split time (h:min)	Cycling split time (h:min)	Running split time (h:min)	Overall race time (h:min)	Transition time (h:min)
Women					
Norway	1:03 ± 0:14	6:48 ± 0:26	4:52 ± 0:21	13:01 ± 0:17	0:06 ± 0:03
Germany	1:10 ± 0:06	7:03 ± 0:28	4:23 ± 0:36	13:46 ± 0:55	0:09 ± 0:03
Great Britain	1:07 ± 0:15	8:06 ± 1:01	6:12 ± 0:43	15:51 ± 1:19	0:14 ± 0:08
Men					
Norway	1:03 ± 0:07	5:34 ± 0:12	4:14 ± 0:12	10:56 ± 0:14	0:03 ± 0:01
Great Britain	1:07 ± 0:08	6:26 ± 0:22	4:57 ± 0:14	12:36 ± 0:41	0:06 ± 0:03
Germany	1:03 ± 0:05	6:28 ± 0:26	4:40 ± 0:16	12:20 ± 0:22	0:08 ± 0:04
France	1:02 ± 0:13	6:33 ± 0:31	5:03 ± 0:34	12:47 ± 0:49	0:08 ± 0:02
Sweden	1:04 ± 0:11	6:24 ± 0:32	4:50 ± 0:25	12:27 ± 1:01	0:08 ± 0:04
Denmark	1:05 ± 0:11	6:35 ± 0:24	4:51 ± 0:40	12:39 ± 1:02	0:08 ± 0:04
USA	1:05 ± 0:05	6:59 ± 0:32	5:30 ± 0:35	13:43 ± 1:08	0:09 ± 0:03
The Netherlands	1:03 ± 0:06	6:53 ± 0:28	5:15 ± 0:41	13:19 ± 0:54	0:08 ± 0:04
Austria	1:04 ± 0:07	6:49 ± 0:30	5:09 ± 0:46	13:14 ± 1:16	0:11 ± 0:04
Belgium	1:03 ± 0:06	6:34 ± 0:15	5:28 ± 0:19	13:13 ± 0:28	0:07 ± 0:03
Spain	1:14 ± 0:09	7:18 ± 0:32	5:29 ± 0:31	14:15 ± 0:59	0:12 ± 0:04

For men, Norwegian men were the fastest in split and transition times although French athletes were the fastest in swimming (Table 6). In swimming, no statistical significant differences were found between the countries. In cycling, men from Norway were faster than men from all other countries (*p* < 0.01 to *p* < 0.0001). Triathletes from Sweden, Great Britain, Germany, France, Belgium, and Denmark cycled faster than triathletes from Spain (*p* < 0.05 to *p* < 0.01). For running, men from Norway were faster than men from France, Austria, the Netherlands, Belgium, Spain and USA (*p* < 0.05 to *p* < 0.0001). German athletes were running faster than triathletes from Belgium, Spain, and USA (*p* < 0.05 for all). Regarding overall race times, athletes from Norway were faster than athletes from all other countries (*p* < 0.05 to *p* < 0.0001). German athletes were faster than American athletes (*p* < 0.05) and athletes from Germany, Sweden, Great Britain, Denmark and France were running faster than athletes from Spain (*p* < 0.05 to *p* < 0.0001). For transition times, athletes from Norway were faster than athletes from USA, Austria, and Spain (*p* < 0.05 to *p* < 0.0001). Athletes from Great Britain (*p* < 0.01) and Belgium (*p* < 0.05) changed faster than athletes from Spain.

**Fig. 2:**
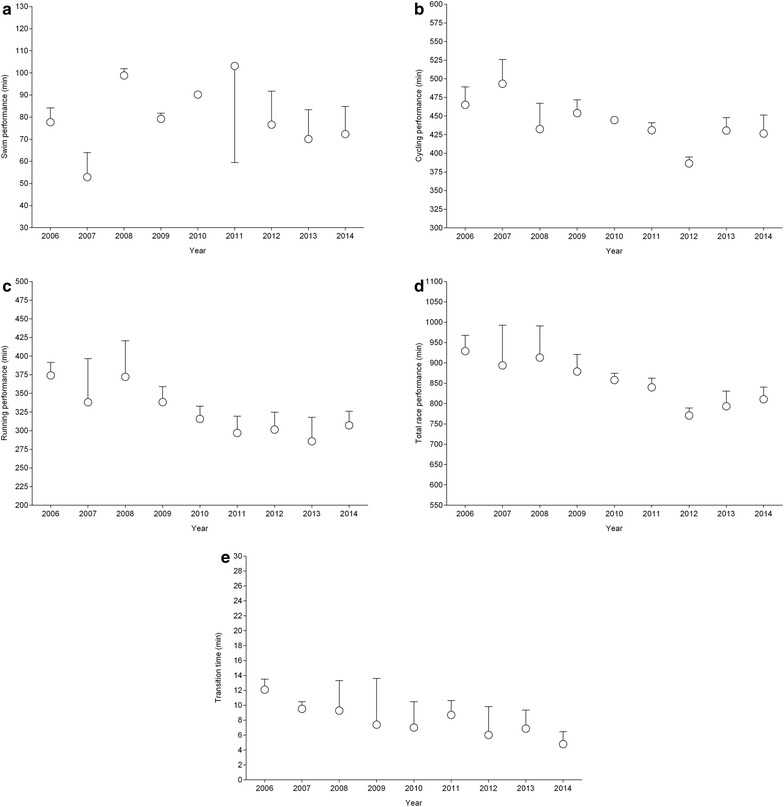
Performance of the annual three fastest Norwegian women in swimming (**a**), cycling (**b**), running (**c**), overall race time (**d**) and transition times (**e**)

**Fig. 3:**
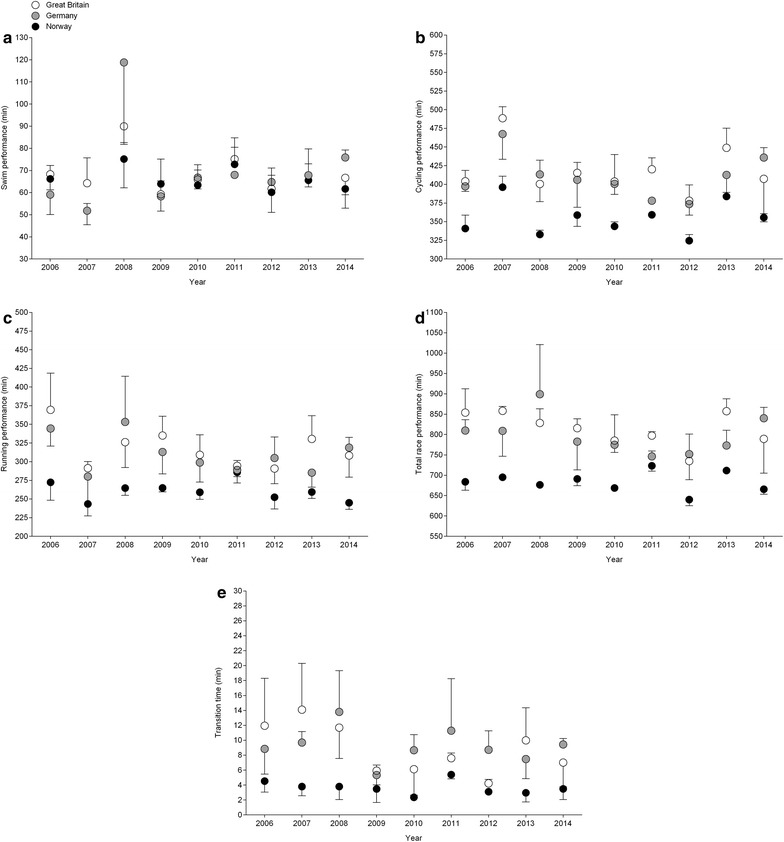
Performance of the annual three fastest men from Norway, Great Britain and Germany in swimming (**a**), cycling (**b**), running (**c**), overall race time (**d**) and transition times (**e**)

Across years, the annual three fastest Norwegian women improved in cycling, running, overall race time and transition times (Fig. 2). In men (Fig. 3), no changes in split times, overall race times or transition times were found for the annual three fastest Norwegians and Germans. British men, however, improved running split times and transition times.

## Discussion

This study investigated participation and performance trend regarding the nationalities of athletes competing in ‘Norseman Xtreme Triathlon’ and it was hypothesized that the race would be dominated by Scandinavian athletes regarding both participation and performance. The most important findings were (1) most of the finishers originated from Norway, (2) the fastest women and men originated from Norway, and (3) the best Norwegian women improved performance across years, but not the best Norwegian men.

### Norwegians dominated participations

As hypothesized, local athletes were the most numerous in ‘Norseman Xtreme Triathlon’. Although this race is considered as one of the toughest triathlon races in the world, local Norwegian women and men dominated participation. This is in line with recent findings for ‘Ironman Hawaii’ where US-American athletes were the most numerous (Dähler et al. 2014). Both ‘Norseman Xtreme Triathlon’ and ‘Ironman Hawaii’ have common that those people who invented the race also competed most frequently. For longer triathlon race distances, however, the trends were differently. US-Americans were the first to start a Double Iron ultra-triathlon (Lenherr et al. 2012), but European athletes are dominating nowadays this kind of race (Rüst et al. 2014a; Sigg et al. 2012).

Holding a race in the own country might also lead to a home advantage. For example, Nevill and Balmer (2012) predicted for the London 2012 Olympic Games a total of 63 medals for Great Britain. Indeed, British athletes won 65 medals. Similarly, a home advantage has been reported for judo. Athletes from countries hosting competitions using the ranking system of the International Judo Federation had an advantage regarding their Olympic classification (Ferreira Julio et al. 2013).

Regarding the finishes, Norwegians were dominating most probably due to the vicinity of the race. Considering recent studies that athletes from neighbouring countries would also compete very frequently, the high participation of athletes from Great Britain is not astonishing. However, British athletes compete more frequently in ‘Norseman Xtreme Triathlon’ than other Scandinavian athletes such as athletes from Sweden, Finland and Denmark. A potential explanation could be that long-distance triathlon is not very popular in these Scandinavian countries and other sports such as winter sports (e.g. cross-country skiing, etc.) would be more popular. An interesting aspect is that athletes from neighbouring countries such as Sweden and Finland were not among the most numerous athletes. We might assume that other sports are more popular in these countries. For example, in Sweden, the most popular sports are soccer, ice hockey, rugby, motor sports and tennis (Most popular sports in Sweden 2015). In Finland, ice hockey, soccer, rugby, martial arts and cycling are the most popular sports (Most popular sports in Finland 2015).

Reasons for a low participation of US-American athletes in ‘Norseman Xtreme Triathlon’ could be financial aspects such as prize money and travel costs. Overall prize money in ‘Ironman Hawaii’ is $ 650,000 (Ironman World Championship 2015). In ‘Norseman Xtreme Triathlon’, the title sponsor offers $ 10,000 for the winner. However, the winner will not get to keep the prize money. The winner gets the T-shirt but the prize money has to be donated to a charity of choice (Norseman Xtreme Triathlon 2015). An US-American athlete intending to compete in Norway would need to pay the flight costs, travel from the airport to the race site, accommodation and entry fee. The relatively high costs may discourage US-American athletes to compete in ‘Norseman Xtreme Triathlon’.

### Norwegians were the fastest

An expected finding was that Norwegian women and men were the fastest in ‘Norseman Xtreme Triathlon’. This is in accordance with ‘Ironman Hawaii’ where also local athletes (i.e. US-American athletes) were the fastest (Dähler et al. 2014). A most likely explanation is that Norwegians are used to their climate. In summer, the average temperature in Norway ranges from 13 to 18 °C (The climate of Norway 2015) and the water temperature in Hardangerfjord is not higher than 16–17 °C. In Hawaii, the climate is tropical with daily highs around 30 °C throughout the month, exceeding 32 °C or dropping below 28 °C only 1 day in 10 (National Weather Forecast Service Office Honolulu 2015). The Pacific Ocean reaches its highest temperature in September and October. The water during winter at Hawaii’s beaches typically maintains a steady 24 °C (Average Ocean Temperatures for Hawaii 2015).

‘Ironman Hawaii’ and ‘Norseman Xtreme Triathlon’ might be considered for long-distance multi-sports athletes like the Olympic Games for athletes competing in other sports disciplines. In the Olympic Games, medals are the primary objective for participating athletes and in Olympic Games it is a measurable test of a nation’s athletic power (Seiler 2013). Considering Norwegian athletes competing in the Olympic Games, they are characterized for a high concentration of results in a few sports disciplines (Seiler 2013).

Among the three fastest were also women and men from Germany and Great Britain when we considered the fastest finishers ever. Similar findings have been reported for ‘Ironman Hawaii’ where British women were second behind US-American women and German men second behind US-American men (Dähler et al. 2014). Most probably both British and German athletes are also used to rather low temperatures. In contrast, US-American women were on 5th position and US-American men on 7th position for the ten fastest times ever in ‘Norseman Xtreme Triathlon’. US-Americans are most probably not used to the harsh climate in ‘Norseman Xtreme Triathlon’.

Regarding performance, split times of the three disciplines decreased throughout the years and the discipline having more impact on overall race time was cycling. Similarly in ‘Ironman Hawaii’, both women and men improved split times across years (Lepers 2008; Rüst et al. 2014b). And generally in long-distance triathlon, cycling and running were more predictive for overall race time than swimming (Knechtle et al. 2007; Lepers et al. 2011).

### The best Norwegian women improved performance across years

An interesting finding was that Norwegian women improved race times, but not Norwegian men. Interestingly, the same finding was made in ‘Ironman Hawaii’ where US-American women improved race times, but not US-American men (Dähler et al. 2014). Social reasons might explain these differences. Women started later in ‘Ironman Hawaii’ than men (The Ironman Story 2015) and it took them longer to stabilize their split and overall race times (Lepers 2008). The same was in ‘Norseman Xtreme Triathlon’ where women started to compete 1 year later than men (Norseman Xtreme Triathlon 2015). There might also be differences in motivation between female and male ultra-endurance athletes (Krouse et al. 2011; Schüler et al. 2014). Women were slower than men, but women reduced this gender gap year after year and above all in the year 2007. Generally, women reduced the gap to men in long-distance triathlons (Lepers 2008; Rüst et al. 2014b). However, in recent years, the gap between women and men remained at 10–20 % for split and overall race times (Rüst et al. 2012b, 2014b).

Although overall race time decreased every year, athletes competing in ‘Norseman Xtreme Triathlon’ have a high potential for injuries. A 26-week prospective cohort study including 174 participants of the 2011 ‘Norseman Xtreme Triathlon’ showed that the average prevalence of overuse problems was ~56 % (Andersen et al. 2013). The average prevalence of substantial overuse problems was ~20 %. The most prevalent sites of overuse problems were the knee (~25 %), the lower leg (~23 %) and the lower back (~23 %). The acute injury incidence was ~0.97 injuries per 1000 h of training and ~1.02 injuries per 1000 h of competition (Andersen et al. 2013).

### Limitations

This study has some limitations, in that some already known performance-related variables, such as age (Knechtle et al. 2014a, b, Stiefel et al. 2013b), training (Kandel et al. 2014; Knechtle et al. 2010), previous experience (Knechtle et al. 2010; Rüst et al. 2011, 2012a), anthropometry (Kandel et al. 2014; Knechtle et al. 2010), pacing (Wu et al. 2014, 2015), fluid intake (Meyer et al. 2012), food intake (Barrero et al. 2014), and environmental conditions (Del Coso et al. 2014) were not included in the analysis.

## Conclusions

To summarize, during 2006-2014, most of the finishers in ‘Norseman Xtreme Triathlon’ originated from Norway and the best race times were achieved by Norwegian women and men. Norwegian women improved race times across years but not Norwegian men.

In particular, the overall race time decreased every year, above all in the year 2012; women were slower than men, but women reduced this gender gap year after year and above all in the year 2007. Athletes from Norway and Germany were faster than those coming from Great Britain and other countries. Split times of the discipline decreased throughout the years. In particular, the discipline having more impact on the final total time was cycling.
